# Motif co-regulation and co-operativity are common mechanisms in transcriptional, post-transcriptional and post-translational regulation

**DOI:** 10.1186/s12964-015-0123-9

**Published:** 2015-12-01

**Authors:** Kim Van Roey, Norman E. Davey

**Affiliations:** Structural and Computational Biology Unit, European Molecular Biology Laboratory (EMBL), 69117 Heidelberg, Germany; Health Services Research Unit, Operational Direction Public Health and Surveillance, Scientific Institute of Public Health (WIV-ISP), 1050 Brussels, Belgium; Conway Institute of Biomolecular and Biomedical Sciences, University College Dublin, Dublin 4, Ireland

**Keywords:** Motifs, Cis-regulatory elements, RNA motifs, Short linear motifs, SLiMs, Co-regulation, Co-operativity, Regulation, Modularity

## Abstract

A substantial portion of the regulatory interactions in the higher eukaryotic cell are mediated by simple sequence motifs in the regulatory segments of genes and (pre-)mRNAs, and in the intrinsically disordered regions of proteins. Although these regulatory modules are physicochemically distinct, they share an evolutionary plasticity that has facilitated a rapid growth of their use and resulted in their ubiquity in complex organisms. The ease of motif acquisition simplifies access to basal housekeeping functions, facilitates the co-regulation of multiple biomolecules allowing them to respond in a coordinated manner to changes in the cell state, and supports the integration of multiple signals for combinatorial decision-making. Consequently, motifs are indispensable for temporal, spatial, conditional and basal regulation at the transcriptional, post-transcriptional and post-translational level. In this review, we highlight that many of the key regulatory pathways of the cell are recruited by motifs and that the ease of motif acquisition has resulted in large networks of co-regulated biomolecules. We discuss how co-operativity allows simple static motifs to perform the conditional regulation that underlies decision-making in higher eukaryotic biological systems. We observe that each gene and its products have a unique set of DNA, RNA or protein motifs that encode a regulatory program to define the logical circuitry that guides the life cycle of these biomolecules, from transcription to degradation. Finally, we contrast the regulatory properties of protein motifs and the regulatory elements of DNA and (pre-)mRNAs, advocating that co-regulation, co-operativity, and motif-driven regulatory programs are common mechanisms that emerge from the use of simple, evolutionarily plastic regulatory modules.

## Background

The life of a gene product, from transcription to degradation, is controlled by a series of regulatory decisions. How does the cell decide when to make a transcript? Does a transcript get translated, stored, decayed or transported to a specific subcellular location? After translation, where is a protein localised, and what complexes should it join? Ultimately, when is a protein degraded? The outcome of this decision-making process is cell state dependent and, consequently, requires the integration of vast amounts of information that is encoded in the local abundance and functional state of a multitude of biomolecules acting as cell state sensors and transmitters. Recent advances in our understanding of cell regulation have suggested that a substantial portion of the interactions that facilitate conditional and dynamic cellular decision-making in higher Eukaryotes are mediated by compact and degenerate interaction modules known as motifs (short linear motifs (SLiMs) in proteins, RNA motifs in RNA and regulatory elements in DNA) [1–5]. The term motif denotes a repeated figure or design and, in motif biology, the occurrence of a given class of motif in a set of unrelated biomolecules led to the appropriation of the term to refer to a recurrent pattern of nucleotides or amino acids that corresponds to an autonomous functional module.

The higher eukaryotic cell has an extensive repertoire of DNA, RNA and peptide motifs that function as dynamic binding modules in complex formation, recruiters of basal regulatory pathways, or receivers of cell state information through association with or modification by their interaction partner [6–8]. These motifs control many aspects of transcriptional (recruiting the basal transcription machinery and transcriptional regulators to the numerous promoters, enhancers, silencers and insulators [6, 9–12]), post-transcriptional (controlling protein production by modulating pre-mRNA splicing; mRNA stability, storage and localisation; and microRNA (miRNA) recruitment [7, 13–17]) and post-translational regulation (controlling a protein’s stability, localisation, modification state and complex association [1, 8, 18, 19]) (Table 1). The regulatory regions of most genes, (pre-)mRNAs and proteins have extensively exploited the available motif repertoire [8, 20, 21] and each biomolecule contains a distinct set of motifs that encode unique regulatory programs tuned to govern the life cycle of the biomolecule [22]. These motifs often occur with high densities as the compact footprint of sequence motifs allows multiple functional modules to be encoded in a short polypeptide or polynucleotide segment [2, 4, 5, 23, 24].

**Table 1:** Representative examples of protein, RNA and DNA motifs

Motif type	Example motif	Consensus sequence^a^	Function
Protein short linear motifs			
Ligand - promote complex formation	SH3 ligand	PxxPx[KR]	Complex formation with SH3 domains [195]
	Nuclear receptor box	LxxLL	Complex formation with Nuclear receptors [196]
	LD motif	[LV][DE]x[LM][LM]xxL	Complex formation with FAT domains [197]
	LxCxE motif	[IL]xCxE	Complex formation with Rb [198]
	RGD motif	RGD	Complex formation with Integrin family members [199]
Localisation - recruit targeting and transport pathways to control protein localisation	Nuclear Export Signal (NES)	ΦxxΦxxxΦxxΦxΦ	Translocation from the nucleus to the cytoplasm [200]
	KDEL ER retrieval signal	[KH]DEL_-COOH_	Translocation from the Golgi to the endoplasmatic reticulum (ER) [201]
	Ciliary targeting signal	RVxP	Transport to the plasma membrane of the cilia [202]
	Peroxisomal targeting signal	[KRH]xxΦ$ or [KRH]Φ$	Import into the peroxisomal lumen [203]
	Tyrosine endocytic signal	YxxΦ	Directs endocytosis of membrane proteins [204]
Enzyme recruitment - recruit enzymes to the protein/complex to modify/demodify a site distinct from the bound motif	Cyclin docking motif	[RK]xLx_{0,1}_[LF]	Recruitment of the Cyclin-Cdk holoenzyme [205]
	PP1 docking motif	RVxF	Recruitment of the PP1 phosphotase holoenzyme [206]
	Tankyrase docking motif	Rxx[PGAV][DEIP]G	Recruitment of the Tankyrase poly-(ADP-ribose) polymerase [207]
	USP7 docking motif	PxxS	Recruitment of the USP7 deubiquitylating enzyme [208]
	NEDD4 docking motif	PPxY	Recruitment of the NEDD4 ubiquitylating enzyme [209]
Stability - recruit E3 ubiquitin ligases and promote substrate polyubiquitylation to control protein stability	APC/C D box degron	RxxLxxΦ	APC/C E3 ubiquitin ligase [210]
	PIP degron	Φ[ST]D[FY][FY]xxx[KR]	Recruitment of the Cdt2 CRL4 E3 ubiquitin ligase [211]
	Fbw7 degron	_p_TPxx_p_[ST]	Recruitment of the Fbw7 SCF E3 ubiquitin ligase [212]
	Oxygen dependent VHL degron	[IL]A_o_Px_{6,8}_ΦxΦ	Recruitment of von Hippel-Lindau protein (pVHL) containing E3 ubiquitin ligase [213]
	MDM2 degron	FxxxWxxΦ	Recruitment of the MDM2 ubiquitin ligase [214]
Modification - act as sites of moiety attachment/removal, isomerisation or cleavage	PIKK phosphorylation site	([ST])Q	Phosphorylation by PIKK family kinases [215]
	Pin1 isomerisation site	_p_[ST](P)	Isomerisation by the Pin1 phosphorylation-dependent prolyl isomerase [216]
	N-Glycosylation site	Nx([ST])	Glycosylation by Oligosaccharyltransferase [217]
	Caspase-3 and −7 cleavage motif	[DE]xxD|[AGS]	Cleavage by Caspase family proteases [218]
	Myristoylation site	_NH2-_M(G)xxx[AGSTCN]	Myristoylation by Myristoyl-CoA:protein N-myristoyltransferase [219]
RNA motifs			
Stability	Adenosine and uridine (AU)-rich elements (ARE)	AUUUA	Recruits positive and negative regulators of mRNA stability [13]
Splicing	5′ splice junction	AG/GURAGU	Recruits splice site recognising U1 snRNA component of the spliceosome [14]
Modification	Polyadenylation signal	AUUAAA	Recruits cleavage and polyadenylation specificity factor (CPSF) to cleave and polyadenylate 3′-UTRs [15]
Localisation	Muscleblind binding motifs	YGCUKY	Targets mRNAs to membranes [16]
miRNA recruitment	miR-125b miRNA response element	CUCAGGG	Regulates expression of mutiple proteins [17]
DNA regulatory elements			
Basal machinery recruitment	TATA box	TATAAAA	Recruitment of the basal transcription machinery to the core gene promoter required for initiation of transcription [9]
Promoters/Enhancers	CCAAT/enhancer binding protein (C/EBP) site	CCAAT	Promotion of gene expression [10]
Silencers/Insulators	CCCTC-binding factor (CTCF) binding site	CCGCGNGGNGGCAG	Diverse functions including acting as a transcriptional repressor and insulator [11]
Endonucleases	EcoRI restriction site	G|AATTC	Sequence specific cleavage of DNA [12]

^a^Patterns are representative and roughly define the specificity of the motif binding partner. Pattern syntax for proteins: letters denote a specific amino acid; “x” denotes any amino acid; square brackets denote a subset of allowed amino acids; curly brackets denote length variability; round brackets indicate a position targeted for post-translational modification after motif recognition; “_p_” denotes a phosphorylation site required for binding; “_o_” denotes a hydroxylation site required for binding; “|” denotes a cleavage site; “Φ” (phi) denotes a aliphatic residue; “_NH2-_” indicates the amino-terminus of the protein; “_-COOH_” indicates the carboxyl-terminus of the protein. Pattern syntax for DNA and RNA: “/” denotes a splice site. “K” denotes a guanine or a uracil; “Y” denotes an adenine or a cytosine; “R” denotes an adenine or a guanine; “N” denotes any base; “|” denotes a cleavage site

Experimental and bioinformatics studies are beginning to offer an insight into the mechanisms driving motif acquisition [4, 25–34]. Many instances are undoubtedly the product of duplication or recombination [25, 31, 35–37]. Conversely, substantial indirect evidence from the comparison of motif presence in different species suggests that motifs can be gained and lost relatively rapidly in homologous regions [26, 27, 31, 34, 38–41]. This observed evolutionary plasticity, in association with their degenerate nature and the limited number of affinity- and specificity-determining residues in a motif, led to the hypothesis that novel motif instances are often acquired through *ex nihilo* motif evolution by point mutations, insertions or deletions [27, 31, 32, 42]. However, catching evolution in the act is difficult. For SLiMs, a serine to glycine mutation in Leucine-rich repeat protein SHOC-2 (SHOC2), which results in a novel myristoylation motif and causes aberrant SHOC2 localisation, provides the sole experimentally characterised example of *ex nihilo* motif birth on the protein level [42]. The mutation is found in several patients with Noonan-like syndrome and for some, the sequence variation is present in neither parents. Thus, the birth of this novel motif is often the result of a germline mutation. A similar mechanism of *ex nihilo* motif acquisition has been hypothesised for nucleotide motifs [31–33]. Indeed, the probability of a motif occurring by chance at a given position is equivalent for the motifs of the three major classes of biomolecule. Consequently, though the three major types of motif are physicochemically distinct they share a similar evolutionary plasticity that has resulted in the ubiquity that gave them their shared name.

The human proteome contains thousands of motif-binding proteins. The current census of nucleotide motif-binding proteins stands at ~1400 DNA-binding proteins [43] and ~850 RNA-binding proteins [44]. The number of SLiM-binding proteins remains to be elucidated, however, given the distribution of known SLiM-binding and -modifying domains in the human proteome, it is likely to be in a similar range [8, 45]. This would suggest that upwards of 20 % of the human proteome might consist of motif-binding proteins. Furthermore, ~2000 human RNA motif-recognising miRNAs have been annotated [46]. Hundreds of distinct classes of motifs recognised by motif-binding biomolecules have been characterised to date [6–8]. The simplicity of motif acquisition has driven the proliferation of motifs of widespread utility and, for several motif classes, experimentally characterised motif instances are present in tens of biomolecules [6, 8, 47]. For a handful of classes, hundreds, or even thousands, of motif instances are known [11, 48, 49]. On the protein level, the high motif density of well-characterised biomolecules [23], the extensive regions of intrinsic disorder [50] (where SLiMs are the predominant functional module type [1, 51]) and the numerous SLiM-binding domains [45] suggest extensive motif use in complex organisms. Recently, Tompa et al. hypothesised that the human proteome may contain up to a million SLiMs [22], however, the actual number of motifs is unknown. The reason is simple, SLiM discovery is difficult: computational approaches have high false positive rates and experimental techniques must overcome the transience of SLiM-mediated interactions, extensive SLiM co-operativity, redundancy and weak phenotypes [52]. However, recent advances in experimental discovery techniques, particularly high-throughput discovery methods, will hopefully rectify this in the coming decade [53].

In this review, while focusing on SLiMs, we aim to highlight the similarities in the use of motif co-regulation and co-operativity in transcriptional, post-transcriptional and post-translational regulation. We discuss how the evolutionary plasticity of sequence motifs facilitated their proliferation and supported the evolution of extensive networks of co-regulation. We examine how the ability to readily add a functional module without disturbing a pre-existing regulatory interface promotes high functional density and how motifs can functionally modulate each other to create decision-making interfaces capable of integrating cell state information. Finally, we consider how multiple motif-containing interfaces in the same biomolecule collaborate to create unique regulatory programs.

### Motif co-regulation

Data from genome sequencing projects has failed to reveal the anticipated correlation between biological complexity and proteome size [54]. This led to the hypothesis that the emergence of increasingly complex organisms was facilitated by an increase in regulation rather than protein number [55–58]. But what supports the increased complexity of regulation in the higher eukaryotic cell?

**Fig. 1:**
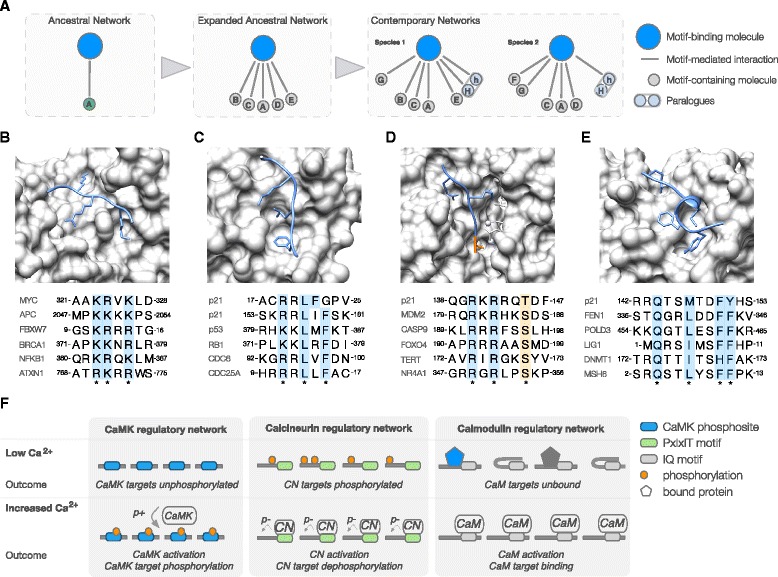
Motif-dependent co-regulation of proteins. **a** Schema showing the expansion of a regulatory network. The original ancestral network will likely contain a limited number of targets. Proteins can be added to the network as they acquire the necessary motifs through *ex nihilo* evolution of novel motifs. Different species will have different regulatory networks [26, 28–30, 122, 123]. **b** Representative motif used to perform basal functionality. Importin-alpha bound to a nuclear localisation signal (NLS)-containing peptide from Myc [124] and representative examples of NLS motifs [125–130], showing the shared residues complementary to the binding pocket (side chains shown in structure) that result in the consensus sequence. **c** Representative motif involved in conditional transmission of cell state information to the motif-containing protein. Cyclin-A2 bound to a Cyclin docking motif in Cellular tumor antigen p53 [131] and representative examples of Cyclin docking motifs [131–135]. **d** Representative motif involved in conditional transmission of cell state information to the motif-containing protein. PKB beta bound to a PKB phosphorylation site peptide from Glycogen synthase kinase-3 beta [136] and representative examples of PKB phosphorylation sites [137–141]. The modified residue is shown in orange. **e** Representative motif used to recruit variable components to an invariant complex core. The PIP box-binding pocket of PCNA bound to a PIP box from p21 [142] and representative examples of PIP boxes [142–147]. **f** Examples of conditional motif-driven regulatory networks in which motifs underlie the co-regulation of multiple biomolecules in a coordinated manner to respond to changes in Ca^2+^ levels. Increased Ca^2+^ levels can result in motif-dependent phosphorylation (p+), dephosphorylation (p-) or competitive binding events (calcium/calmodulin-dependent protein kinase (CaMK) recognises Rxx[ST] [64], Calcineurin (CN) phosphatase recruits substrates through PxIxIT or LxVP docking motifs [65], and Calmodulin (CaM) recognises hydrophobic helical IQ motifs [66])

One key feature of eukaryotic regulation is the extensive reuse of specialised regulatory pathways. The ease of motif acquisition, facilitated by their evolutionary plasticity, makes them the ideal module to simplify access to systems of widespread utility, and evolution appears to have exploited this extensively. Accordingly, many motifs encode the ability to recruit components of these regulatory systems (Table 1). The intrinsic evolutionary properties of motifs have facilitated the evolution of large networks of biomolecules that bind to a single motif-binding hub acting as recognition element for the regulatory machinery (for instance, gene promoters containing hypoxia response elements (HREs) recruit the HIF-1 complex to induce expression of genes involved in the response to limited oxygen conditions [59]; co-regulation of the translation and stability of mRNAs encoding proteins involved in iron metabolism by iron-responsive elements (IREs) in the untranslated regions (UTRs) that bind iron regulatory proteins depending on iron availability [60]; concerted degradation of cell cycle regulatory proteins in a cell cycle phase-dependent manner through recognition of specific degron motifs by the Anaphase-Promoting Complex/Cyclosome (APC/C) ubiquitin ligase [61]). As a result, instances of the same motif class are regularly present in multiple distinct biomolecules [8, 30, 48, 62] (a motif class defines the set of motifs that recognise a single motif-binding pocket on a specific biomolecule). Interestingly, these networks are evolutionarily dynamic and differ between even closely related species [27, 41, 63]; however, it appears that once a functionally valuable motif-accessible system is in place, additional biomolecules come under the control of these systems, thereby extending the regulatory networks (Fig. 1a) [48]. Most of the more abundant motifs link biomolecules to the molecular machinery that performs important basal house keeping functions. Basal functions can be required by thousands of biomolecules and consequently many of the motifs that facilitate these functions are ubiquitous (for example, the motifs that recruit the basal transcription, splice site recognition and protein translocation machinery [48, 49, 62]) (Fig. 1b). An important subset of the regulatory machinery is the conditionally, temporally or spatially restricted motif-binding molecules that transmit cell state information to the motif-containing biomolecule (Fig. 1c and d). The cell contains numerous motif-accessible pathways that allow biomolecules to integrate cell state information in their interfaces to respond appropriately and in a coordinated manner to changes in their environment (for example, fluctuations in calcium levels [64–66] (Fig. 1f), transitions of cell cycle phase [41, 67–69] or detection of DNA damage [70, 71]). On the protein level, motif-binding pockets can also recruit several distinct motif-containing regulatory proteins to a complex. In these cases, the motif facilitates the construction of functionally distinct assemblies around a constant complex core, for example, the recruitment of PIP box motif-containing proteins to the DNA sliding clamp by Proliferating cell nuclear antigen (PCNA) [72, 73] (Fig. 1e), the recruitment of SxIP motif-containing proteins to microtubule plus-end binding proteins [74], or the recruitment of LxCxE motif-containing proteins to E2F-regulated promoters by Retinoblastoma-associated protein (Rb) [75].

Thus, the evolutionary properties of motifs simplify access to many, widely relevant functionalities and facilitate the construction of diverse functional assemblies around a constant complex core. The higher eukaryotic cell contains innumerable co-regulated networks of biomolecules that are connected by motifs. Experimental analyses of these networks should consider that the modulation of a single motif could have effects across the network.

### Motif co-operativity

Motifs are autonomous functional binding modules that can independently engage in an interaction. Many motifs can function in isolation, however, in many cases, a binding or modification event at one motif will affect binding to or modification of another motif, i.e. motifs generally act co-operatively. Multiple distinct motif-mediated binding and/or modification events can affect each other either positively or negatively to various degrees, i.e. they can induce, promote, inhibit or completely abrogate each other. The cell extensively exploits motif co-operativity and to date, many experimentally validated cases of co-operative binding of motifs have been described [19]. Co-operative binding can serve to increase the specificity of an interaction, to increase the affinity of an interaction, and/or to integrate cell state information, as will be described in the following paragraphs [1, 4].

**Fig. 2:**
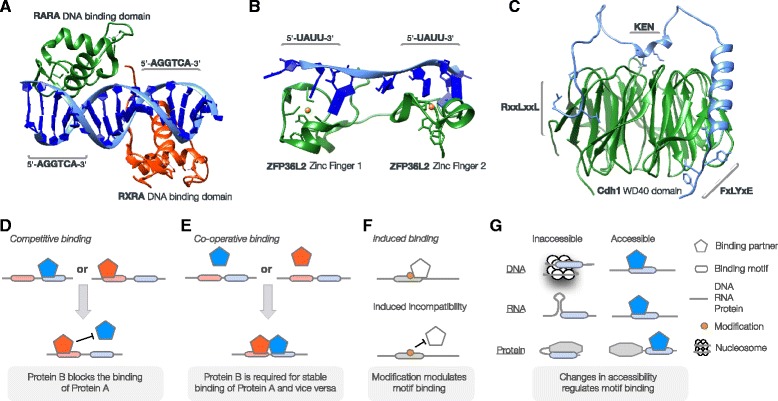
Examples of co-operative interactions mediated by DNA, RNA and protein motifs. **a** DNA motif specificity through multivalent interactions with motif-binding domains in multimeric complexes. Structure of Retinoic acid receptor alpha (RARA) (green) and Retinoic acid receptor RXR-alpha (RXRA) (red) heterodimer bound to a retinoic acid response element (5′-AGGTCAAAGGTCA-3′) (blue) [107]. Each protein binds to a 6-mer “half-site” (5′-AGGTCA-3′) giving the complex specificity for a 12-mer motif. **b** RNA motif specificity through multivalent interactions with tandem arrays of motif-binding domains. Structure of the tandem Zinc Fingers of Zinc finger protein 36, C3H1 type-like 2 (ZFP36L2) (green) bound to an RNA class II AU-rich element (ARE) (5′-UUAUUUAUU-3′) (blue). Each Zinc Finger recognises 4 nucleotides of RNA, allowing the tandem domains to recognise an 8-mer motif [78]. **c** Protein motif specificity through multivalency. Structure of yeast APC/C-Cdh1 modulator 1 (Acm1) (blue) bound to APC/C activator protein Cdh1 (green) showing the 3 binding pockets for the D box (RxxLxxL), KEN box (KEN) and ABBA motif (FxLYxE) on the WD40 repeat of Cdh1 [80]. **d** Example of competitive motif-mediated binding involving two motifs. Binding of a single biomolecule/complex to a motif is sufficient to perform the biological function; however, when a second biomolecule is present, the function facilitated by the first site is inhibited [19, 87, 148–150]. **e** Schematic example of co-operative motif-mediated interactions involving two motifs. In the example, binding of a single interface is insufficient to elicit the functional outcome of binding. Once the second motif-binding interface associates, the trimeric complex can bind with sufficient affinity/avidity to elicit the biological outcome. **f** Modification on or near a regulatory motif can modulate the motif either positively [89, 151–154] or negatively [18, 19, 94]. **g** Motif accessibility is required for binding partner recruitment and, consequently, is often utilised as a step of regulation [18, 19, 99, 100, 155]

A common strategy in motif interactions is the co-operative binding of multiple motifs and motif-binding domains, which in isolation are somewhat promiscuous, to mediate highly specific interactions. Motif-binding domains or motifs can co-operate at an intermolecular level, through multimerisation of the motif-binding or motif-containing partners [76] (Fig. 2a), or at an intramolecular level, for example many motif-binding domains (e.g. zinc fingers for DNA motifs, RNA recognition motifs (RRM) for RNA motifs, and SH2, SH3 and PDZ domains for SLiMs) occur as tandem arrays to increase binding specificity [77–79] (Fig. 2b). In proteins, multiple pockets on the same globular domain can also function co-operatively [80] (Fig. 2c). These mechanisms, in addition to temporal and spatial separation of biomolecules [81], permit high-fidelity recognition of biologically relevant binding partners despite the large number of sequences that are complementary to the specificity of a single motif-binding module [4]. The same mechanisms also allow the intrinsically weak affinities of a single motif (a particular feature of SLiMs, which mediate interactions with affinities that are generally in the 1–10 μM range) to be increased by binding multivalently with high avidity. The binding strength of these interactions can increase by orders of magnitude while the system retains much of the dynamism of the constituent parts [82, 83]. For instance, robust localisation of Amphiphysin 1 to the periphery of assembling clathrin lattices depends on two distinct motifs that bind to two independent sites on the N-terminal beta-propeller domain of clathrin, which increases the affinity and specificity of the interaction [84]. Similarly, higher order use of co-operative avidity-driven binding mechanisms also allows motifs to recruit, organise and stabilise large dynamic multimeric complexes such as those that assemble at DNA regulatory element-rich gene promoters [24] or on SLiM-rich scaffolding proteins [1, 85].

In addition to directing multi-partite interactions with high specificity and avidity, motif co-operativity also plays a fundamental role in cellular decision-making. A single motif instance is not intrinsically conditional. However, through regulation of the local abundance of the motif-binding partner and/or through co-operative or competitive use of multiple motifs, combinatorial decision-making is possible [1]. A binding or modification event at one motif can modulate the occupancy state of another motif, thus changing the functionality of the second motif. Accordingly, the co-operative nature of their interactions provides motifs the means to integrate cell state information from multiple inputs and propagate regulatory decisions based on this information. Binding motifs can influence each other in different ways [18, 19]. Overlapping or adjacent motifs can promote mutually exclusive, competitive interactions, allowing context-dependent assembly of functionally distinct complexes [86] (Fig. 2d). For instance, in Rb, the docking motif for the catalytic subunit of protein phosphatase 1 (PP1) and the cyclin docking motif that recruits cyclin-Cdk complexes overlap. While binding to PP1 results in dephosphorylation of Rb, keeping it active as a repressor of E2F-dependent transcription, binding to cyclin-Cdk results in phosphorylation and inactivation of Rb, thus promoting cell cycle progression [87]. Alternatively, adjacent motifs can co-operate positively, facilitating the integration of signals encoded in the presence of their different binding partners [88] (Fig. 2e). Such co-operativity occurs during assembly of the T cell signalling complex on the Linker for activation of T-cells family member 1 (LAT) scaffold protein, which contains multiple SH2 domain-binding motifs that, upon phosphorylation, recruit a variety of signalling proteins through their respective SH2 domains to build a functional signalling complex [88]. Another key mechanism for cell state dependent decision-making is mediated by modulation of the intrinsic affinity and/or specificity of a motif by modification of one or more overlapping or neighbouring modification motifs [89, 90]. The binding properties of a motif can be adjusted by the covalent attachment of a moiety (Fig. 2f), ranging from switching on intrinsically inactive motifs that require a specific modification in order to be active [91, 92] (for instance, Plk1-catalysed phosphorylation of two serine residues in the beta-TrCP-binding degron in Claspin is required for its interaction with beta-TrCP and the associated ubiquitin ligase complex, resulting in ubiquitylation and subsequent proteasomal degradation of Claspin, a process involved in termination of the DNA replication checkpoint [93]), disrupting an interaction [94, 95] (such as binding of the USP7-docking motif in Mdm4 to the deubiquitylating enzyme USP7, which is inhibited by phosphorylation of a serine residue adjacent to the motif by ATM kinase to promote Mdm4 destabilisation during DNA damage response [96]) or changing the specificity of a binding region from one binding partner to another [97] (for example, phosphorylation of a tyrosine residue in a PTB domain-binding motif in the Integrin beta-3 tail negatively regulates integrin activation by switching the specificity of the binding region from Talin to Dok1 [98]). The binding properties of a motif or a motif-binding domain can also be modulated indirectly by allosteric effects, resulting from modification or effector association/dissociation at a site that is distinct from the actual interaction interface [99–101] (Fig. 2g). A well characterised example of allosteric regulation of SLiM-mediated interactions involves ligand-induced activation of the Wiskott-Aldrich syndrome protein (WAS), where binding of Cdc42 relieves a motif-mediated auto-inhibitory interaction in WAS, resulting in activation of the protein [102].

On a molecular level, some motifs will function independently, whereas others will be contained in multi-motif co-operative interfaces. This raises the question whether there exist pairings of motifs that can cooperate and others that cannot? Or is the requirements of the system the only limit on the observed co-operative motif pairings? The mechanisms driving the evolution of motif co-operativity is an open question and only a handful of examples of a co-operative motif being added to a pre-existant motif interface have been fully characterized [25, 39]. However, given the simplicity of motif acquisition, most motif pairings will have been tested by evolution. It is likely that unobserved pairings are of limited biological utility and consequently are not retained. It is clear that many commonly observed co-operative motif pairings reflect the available motif-binding pockets in the binding partner, for example, docking motifs and modification sites for the same PTM enzyme will often occur in the same protein, increasing the efficiency and specificity of modification [78, 80, 103–107]. Furthermore, intuitively, motifs with related functionality will be more likely to co-operate (i.e. cell cycle kinase modification motifs often regulate adjacent cell cycle-related interaction motifs such as the mitotic degron motifs [108–111]). Depending on the spatial organisation and flexibility of the motif-binding partner, constraints may be placed on the minimum or maximum inter-motif distance and the ordering of the motifs; such constraints have been observed for the APC/C and the Cdk/Cyclin/Cks1 complex [80, 112–114].

In summary, the unique evolutionary and binding attributes of motifs in DNA, RNA and proteins facilitate two highly exploited mechanisms: (i) the co-operative use of multiple independent low-affinity and low-specificity binding sites to allow highly specific assembly of dynamic, meta-stable complexes, and (ii) the co-operative integration of information in conditional decision-making interfaces. Consequently, the function of many motifs cannot be fully determined if the analysis is restricted to discrete instances.

### Motif-driven regulatory programs

Evolution rarely creates completely new molecular functions, and more readily works with existing tools to produce novelty—as François Jacob stated, “Evolution is a tinkerer, not an inventor” [115]. On the molecular level, this is clearly evident as the modular nature of biomolecules permits evolution to reuse useful modules in novel combinations to produce distinct biological outcomes [116].

**Fig. 3:**
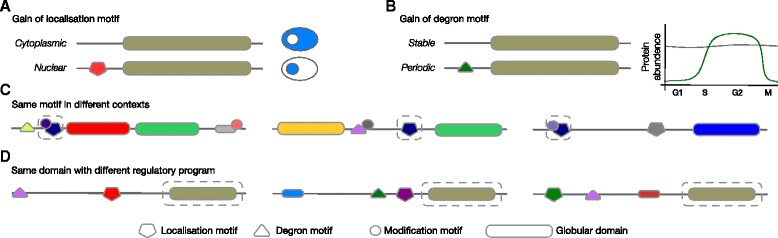
Distinct regulatory programs and protein modularity. **a** The higher eukaryotic cell has a large repertoire of protein modules, represented here by different shapes with different colours, that are reused by evolution to encode many aspects of protein functionality, including its subcellular localisation (pentagons), stability (triangles), modification state (circles) and interactome (rectangles). The *ex nihilo* acquisition of a targeting SLiM can result in protein relocalisation. For instance, while a protein without an NLS motif (top) is expressed ubiquitously throughout the cytoplasm (blue zone), acquisition of an NLS motif (bottom, red pentagon) results in specific localisation of the protein in the nucleus (blue zone). **b** The *ex nihilo* acquisition of a degradation motif can result in changes to the temporal, spatial or conditional local abundance of a protein. For instance, while the abundance of a protein without a cell cycle-specific degron (top) is independent of the different phases of the cell cycle, acquisition of a cell cycle-specific degron (bottom, green triangle), for example a D box motif, allows the abundance of the protein to be adjusted for a specific phase of the cell cycle. **c** Example of co-regulation of a protein by the same motif (boxed blue pentagon). The three different proteins will be regulated in a similar manner under specific conditions through recruitment of the same binding partner by the shared motif, for instance cell cycle-dependent degradation of cell cycle regulators such as Acm1 [156], Cyclin A [157] and Securin [158], which are targeted to the APC/C for ubiquitylation through their D box motifs. **d** Proteins with instances of the same globular domain (boxed brown rectangle) can have hugely different life cycles depending on the set of motifs present in the protein. While the proteins have a similar activity due to the shared globular domain, their distinct motif content subjects them to specific regulatory programs and diversely controls their life cycle, as is the case for the different members of the CDC25 family of phosphatases [117] and the Cyclin-dependent kinase inhibitor family [118]

The cell has a vast repertoire of DNA, RNA and protein motifs that carry out a wide range of functions (Table 1). Addition of these motifs can have a marked effect on a biomolecule; for example, on the protein level, addition of modules can modify the subcellular localisation, stability, modification state and interactome of a protein, hence affecting its activity and function (Fig. 3a–b). The small footprint of motifs permits the addition of a module to add novel functionality without disrupting the ancestral functionality [25, 39]. Consequently, biomolecules can contain multiple motifs [117, 118] (Table 2). As discussed in the previous section, each motif can co-operate with additional motifs and together these simple components can exhibit complex behaviour due to their conditional connectivity. The set of motifs in a biomolecule encodes a regulatory program that defines the logic of its decision-making circuitry: controlling under what conditions and to what degree transcription proceeds; the processing, location, stability and translation of RNA; and the localisation, stability, modification state and interactome of a protein. The regulatory program also defines how the biomolecule integrates the available information encoded in its own local abundance, the local abundance of its binding partners, binding site occupancy and modification state, to produce a functional outcome. Different sets of modules, or the same set of modules with distinct conditional connectivity, can respond differently to the same changes in cell state, allowing each biomolecule to build unique regulatory programs (Fig. 3c–d).

**Table 2:** Representative examples of motifs modulating the abundance and function of Cyclin-dependent kinase inhibitor 1 (p21)

Motif	Motif sequence	Binding domain/partner	Function
Protein short linear motifs			
Cyclin docking motif [187]	_19_RRLF_22_	Cyclin fold of G1/S-specific cyclin-E1	Inhibition of Cyclin E-Cdk2 catalytic activity and substrate recruitment
Cyclin docking motif [188]	_155_RRLIF_159_	Cyclin fold of G1/S-specific cyclin-E1	Docking to the Cyclin E subunit of the Cyclin E-Cdk2 kinase complex, which results in phosphorylation of p21 at S130 by Cdk2 and subsequent destabilisation of p21
PCNA-binding PIP box [86, 186]	_144_QTSMTDFYHS_153_	Proliferating cell nuclear antigen	Inhibition of the DNA polymerase delta processivity factor PCNA, resulting in G1 and G2 cell cycle arrest
Nuclear localisation signal (NLS) [189]	_142_RRQTSMTDFYHSKRRLI_158_	Armadillo domain of Importin-alpha	Translocation of p21 from the cytosol to the nucleus where it exerts it’s effects on cell proliferation
APC/C-binding D Box degron [185]	_86_RDELGGGR_93_	WD40 repeat of Cell division cycle protein 20 homolog	Ubiquitylation of p21, thereby targeting the protein for proteasomal degradation during prometaphase
PIP degron motif [183]	_145_TSMTDFYHSKRRL_157_	WD40 repeat of Denticleless protein homolog	PCNA- and ubiquitin-dependent proteasomal degradation of p21 in S phase and after UV irradiation
Cdk2 phosphosite [193]	_130_(S)P_131_	Kinase domain of Cyclin-dependent kinase 2	Targets p21 for ubiquitylation and subsequent proteasomal degradation
PKB phosphosite [190]	_140_RKRRQ(T)_145_	Kinase domain of Protein kinase B (PKB)	Results in cytoplasmic localisation of p21, prevents complex formation with PCNA, and decreases the inhibitory effect on Cyclin-Cdk complexes
NDR phosphosite [192]	_141_KRRQT(S)_146_	Kinase domain of nuclear-Dbf2-related (NDR) kinases	Destabilisation of p21 protein to control G1/S progression
RNA motifs			
miRNA [119]	miRNA seed region (AAAGUGC) complementary sites within the 3′-UTR	miRNA miR-17,20a, 20b, 93, 106a, and 106b	Down-regulation of p21 expression
HuD binding site [177, 220]	_688_UUGUCUU_695_	RRM domain of ELAV-like protein 4	Increased stability of p21 mRNA
HuR binding site [178, 220]	AU-rich elements within nt 751–850	RRM domain of ELAV-like protein 1	Increased stability of p21 mRNA
RNPC1 binding site [179, 220]	AU-rich elements within nt 621–750	RRM domain of RNA-binding protein 38	Increased stability of p21 mRNA
Msi-1-binding site [180]	_1819_GUAGU_1823_ (on a loop portion of a stem–loop–stem structure)	RRM domain of RNA-binding protein Musashi homolog 1	Inhibition of p21 mRNA translation to regulate progenitor maintenance
GC-rich sequence [148]	within nt 37–59	RRM domain of CUGBP Elav-like family member 1	Increased translation of p21 mRNA
GC-rich stem–loop structure [148]	within nt 37–59	Calreticulin	Blocks translation of p21 mRNA via stabilisation of a stem-loop structure within the 5′ region
CU-rich sequence [181]	CCANNCC within the 3′-UTR	KH domain of Heterogeneous nuclear ribonucleoprotein K	Repression of p21 mRNA translation
DNA regulatory elements			
p53-responsive element [159, 160]	GAACATGTCCCAACATGTT at −2233 and GAAGAAGACTGGGCATGTCT at −1351	Cellular tumor antigen p53	p53-mediated up-regulation of p21 gene transcription in response to stress signals such as DNA damage
E-box motif [161]	CAGCTG at −420, −163, −20 and −5	Helix-Loop-Helix of Transcription factor AP-4	AP-4-dependent repression of p21 gene transcription in response to mitogenic signals
Retinoid X response element (RXRE) [162]	AGGTCAGGGGTGT at −1198 and GAGGCAAAGGTGA at −1221	zf-C4 zinc finger of Retinoic acid receptor RXR-alpha	RXR ligand-dependent induction of p21 gene expression by RXR-alpha
Retinoid acid response element (RARE) [163]	AGGTGAAGTCCAGGGGA at −1212	zf-C4 zinc finger of Retinoic acid receptor alpha (RAR-alpha)	Retinoic acid-dependent induction of p21 gene expression by RAR-alpha
Vitamin D response element (VDRE) [164]	AGGGAGATTGGTTCA at −770	zf-C4 zinc finger of Vitamin D3 receptor	1,25-dihydroxyvitamin D3-dependent induction of p21 gene expression by Vitamin D3 receptor
CDX binding site [167]	Three TTTAT within −471 to −434	Homeobox domain of Homeobox protein CDX-2	Activation of p21 gene transcription by CDX-2
T-element [168]	AGGTGTGA close to the transcription start site (TSS)	T-box of T-box transcription factor TBX2	Repression of the p21 gene promoter by TBX2
STAT binding element [165, 166]	TTCCCGGAA at −647, TTCTGAGAAA at −2541 and CTTCTTGGAAAT at −4183	STAT fold of Signal transducer and activator of transcription (STAT) proteins STAT1/STAT3/STAT5	STAT-dependent activation of p21 gene expression in response to several cytokines
NF-IL6 site [169]	GTACTTAAGAAATATTGAA at approximately −1900	bZIP domain of CCAAT/enhancer-binding protein beta	Induction of p21 gene expression by CCAAT/enhancer-binding protein beta
Sp1 binding site [170–173]	6 GC-rich Sp1-binding sites between −120 and TSS	C2H2 zinc finger of Transcription factor Sp1/Sp3	Sp1/Sp3-dependent induction of p21 gene expression
AP2 binding site [174]	GCGGTGGGC at −103	Transcription factor AP-2-alpha	Induction of p21 transcription and growth arrest by AP-2-alpha
E2F binding site [175]	CTCCGCGC at −155 and CGCGC at −103, −89 and −36	Winged-Helix of Transcription factor E2F1	Activation of the p21 gene at the G1/S boundary by E2F1
Forkhead binding site [176]	TGTGTGC at +200 3′ of TSS	Forkhead domain of Forkhead box protein P3	Induction of p21 transcription by Forkhead box protein P3

**Fig. 4:**
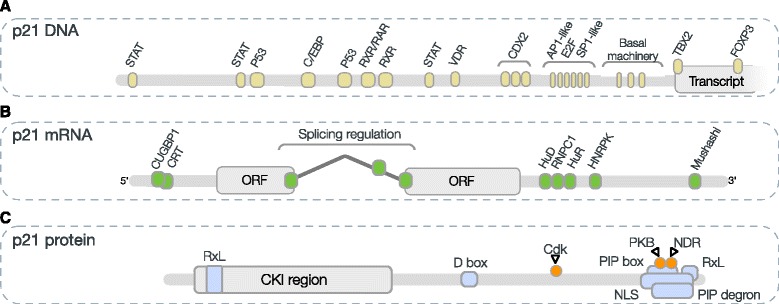
Modular architecture of p21 gene, pre-mRNA and protein, showing known functional modules (see Table 2). **a** The p21 gene contains: two p53-responsive elements [159, 160]; four E-box motifs for binding Transcription factor AP-4 [161]; retinoid X response [162], retinoid acid response [163] and Vitamin D response [164] elements; three STAT-binding elements that recruit STAT1, STAT3 and STAT5 dimers [165, 166]; three CDX-binding sites that bind homeobox protein CDX-2 [167]; a T-element that binds the T-box transcription factor TBX2 [168]; a binding site for CCAAT/enhancer-binding protein beta [169]; six Sp1-binding sites [170–173]; a site for binding Transcription factor AP-2-alpha [174]; sites for Transcription factor E2F1 [175]; a Forkhead-binding site for Forkhead box protein P3 [176]. **b** The p21 (pre-)mRNA contains: AU-rich elements in the 3′-UTR for binding ELAV-like protein 4 [177], ELAV-like protein 1 [178], and RNA-binding protein 38 [179]; a binding site for RNA-binding protein Musashi homolog 1 [180]; GC-rich sequence binding CUGBP Elav-like family member 1 and calreticulin (CRT) [148]; CU-rich sequence in the 3′-UTR for binding heterogeneous nuclear ribonucleoprotein K [181]; splice donor and acceptor site for recruitment of the spliceosome machinery for intron removal. ORF: open reading frame. **c** The p21 protein contains: the intrinsically disordered Cyclin-dependent Kinase Inhibitor (CKI) region [182]; a PIP degron recruiting Denticleless protein homolog [183, 184]; a D box for docking to the Cell division cycle protein 20 homolog subunit of the APC/C [185]; a PIP box for docking to the DNA polymerase delta processivity factor PCNA [142, 186]; one N-terminal and one C-terminal RxL Cyclin docking motif for binding to the Cyclin E subunit of the Cyclin E-Cdk2 kinase complex [187, 188]; an NLS for recruitment to the nuclear import machinery [189]; a modification motif for phosphorylation at T145 by PKB [190, 191]; a modification motif for phosphorylation at S146 by nuclear-Dbf2-related (NDR) kinases [192]; a modification motif for phosphorylation at S130 by Cyclin E-Cdk2 kinase complex [193, 194]

Ultimately, tens to hundreds of modules in DNA, RNA and proteins, many of them motifs, regulate the life cycle of every gene product on the transcriptional, post-transcriptional and post-translational levels from transcription to degradation (Table 2, Fig. 4) [119].

## Conclusions

Biomolecules are robustly regulated from their transcription to their destruction to generate high fidelity control of cell physiology. An emerging concept in biology is that compact functional modules recognised by DNA-binding, RNA-binding and SLiM-binding biomolecules control much of the conditional decision-making in a cell [18, 120, 121]. The three major classes of biomolecules, DNA, RNA and proteins, extensively utilise short sequence motifs to determine the various aspects of their regulatory functionality and to conditionally recruit effectors based on the current cell state. Proliferation of these motifs facilitates biomolecule co-regulation and increases the complexity of cell regulation by expanding existing networks, thereby increasing the density of network wiring without any requirement to add new molecules to the proteome.

The discovery of the complete set of motifs is vital to our understanding of cell regulation. However, motifs co-operate and compete to encode the logic of decision-making and together, co-regulation and co-operativity produce intricate biological outcomes from simple motifs, generating the complicated regulation that underlies higher eukaryotic cell physiology. Consequently, to truly appreciate the regulatory program of a biomolecule, we cannot solely determine the repertoire of motifs, we must also establish the conditional connectivity between motifs. Thus, the regulatory segments of genes, the 5′-UTRs, 3′-UTRs and introns of (pre-)mRNAs, and the intrinsically disordered regions of proteins should be seen as functionally analogous regions, and the DNA regulatory elements, RNA motifs and SLiMs contained within these regions should be considered the cornerstones of regulation in complex organisms, for without them, the observed level of regulatory complexity would not be achievable.

## Abbreviations

SLiMs: Short linear motifs
miRNA: microRNA
HREs: Hypoxia response elements
IREs: Iron-responsive elements
UTRs: Untranslated regions
APC/C: Anaphase-promoting complex/Cyclosome
RRM: RNA recognition motifs
ER: Endoplasmatic reticulum
NES: Nuclear export signal
PKB: Protein kinase B
NLS: Nuclear localisation signal
